# Unveiling the therapeutic profile of pomiferin: a meta-analysis of cytotoxicity and oxidative stress modulation in preclinical models

**DOI:** 10.3389/fphar.2026.1789772

**Published:** 2026-03-23

**Authors:** Burcu Yuksel, Nurullah Eryilmaz

**Affiliations:** 1 Kocaeli University, İzmit, Türkiye; 2 University of Bath, Bath, United Kingdom

**Keywords:** cancer therapy, CR2, *IC*_*50*_, meta-analysis, oxidative stress, pomiferin

## Abstract

**Objective:**

Pomiferin, a prenylated isoflavonoid from *Maclura pomifera*, shows anticancer and antioxidant potential. However, data vary across cell lines and models. This meta-analysis aims to quantify its *IC*_*50*_ in different cancer types and systematically synthesise its effects on oxidative stress biomarkers, thereby establishing a quantitative synthesis for its therapeutic profile.

**Methods:**

A systematic search in PubMed, Scopus, and Web of Science was conducted following PRISMA guidelines. A “mean-only” approach was used to manage within-study dependency and inconsistent variance reporting. Data were analysed with multilevel random-effects models and Cluster-Robust Variance Estimation (CR2).

**Results:**

The pooled geometric mean *IC*_*50*_ for Pomiferin was 12.6 μM (*logIC*_*50*_ = 2.53). Meta-regression showed that efficacy varied by tumour type, with Glioma/neurological models having higher *IC*_*50*_ values (lower efficacy) than others (*p* = 0.012). Pomiferin increased Catalase activity (*p* = 0.044) and protein levels (*p = 0.033*). No statistically significant difference in efficacy was detected between *in vitro* and *in vivo* models (*p = 0.190*); however, current data limitations preclude establishing definitive biological equivalence.

**Conclusion:**

This meta-analysis demonstrates that Pomiferin is a context-dependent agent that modulates enzymatic defences, such as CAT, confirming its potential as a therapeutic candidate. It highlights the efficacy limits in resistant types, such as Glioma, and provides a quantitative basis for future research. This study consolidates diverse findings into a robust evidence base, serving as the first comprehensive meta-analysis of Pomiferin’s biological activity and guiding future studies.

## 1 Introduction

Cancer remains one of the leading causes of morbidity and mortality worldwide, placing an increasing burden on global healthcare systems (Yuksel et al., 2022). Current epidemiological data show that cancer incidence has reached alarming levels. For example, by 2020, it was reported that breast cancer cases had reached 2.26 million, making it the leading cause of cancer-related deaths among women (Rumpa and Maier, 2024). Likewise, gastric cancer continues to be a major public health problem in East Asia, Eastern Europe, and South America, with at least 1 million new cases diagnosed annually (Guang et al., 2024). In paediatric oncology, neuroblastoma (NB) is notable for its aggressive course, as it is the most common extracranial solid tumour in early childhood (Gnanamony et al., 2025).

Despite advancements in surgical interventions, radiotherapy, and the use of chemotherapeutic agents, success rates in cancer treatment remain limited due to tumour heterogeneity, metastasis, and, most significantly, multidrug resistance (MDR) mechanisms. The complexity of the tumour microenvironment and the ability of cancer cells to evade apoptosis (“cell death resistance”) diminish the efficacy of standard therapies (Qu et al., 2023). Resistance developing specifically against first-line chemotherapeutics such as cisplatin, doxorubicin, and taxol is associated with the overexpression of ATP-binding cassette (ABC) transporters, such as P- glycoprotein (P-gp/MDR1), or the downregulation of pro-apoptotic proteins (BAK, BAX). Furthermore, the severe side effects of existing cytostatic drugs—including nephrotoxicity, neurotoxicity, and emetogenesis—reduce patients’ quality of life and lead to dose-limiting toxicities (Vančo et al., 2021). Consequently, this necessitates the discovery of novel therapeutic agents that possess higher selectivity, minimised side effects, and the capability to specifically target resistant tumour phenotypes.

Natural compounds have historically been a vital resource in drug discovery. Plant-derived phytochemicals, long used in traditional medicine, are being reassessed in modern pharmacology for their chemopreventive and chemo-adjuvant properties (Betts et al., 2023). In this context, Pomiferin—a prenylated isoflavonoid obtained from the fruits of *Maclura pomifera* (Osage orange)—has emerged as a promising candidate in recent *in vitro* and *in vivo* studies. The literature often highlights that Pomiferin not only exhibits selective cytotoxicity against cancer cells but also protects tissue by regulating oxidative stress (Gruber et al., 2014; Son et al., 2007). Although its fruits are not edible, they are rich in bioactive phytochemicals, especially the prenylated isoflavonoids Pomiferin and Osajin. First isolated in 1939, these two compounds share a similar scaffold but differ in biological efficacy due to their structural differences (Gruber et al., 2014). The enhanced efficacy of Pomiferin (*C*_*25*_ *H*_*24*_ *O*_*6*_) is attributed to the catechol moiety (dihydroxylated B-ring) at the 3′ and 4′ positions, while Osajin has only a single hydroxyl group. Comparative studies indicate that this structural variation gives Pomiferin significantly higher antioxidant and cytoprotective properties (Da Silva et al., 2022).

Given the significant variability in Pomiferin’s bioactivity reported in the current literature, a systematic synthesis of available data is necessary to reconcile fragmented qualitative observations. This study presents a comprehensive meta-analysis, conducted in accordance with PRISMA guidelines, to assess Pomiferin’s therapeutic efficacy across various malignancies. By quantifying *IC*_*50*_ values and evaluating its effects on oxidative stress biomarkers (e.g., SOD, MDA, GSHPx), we aim to move from dispersed mechanistic findings to a unified quantitative model of its action. To ensure the robustness of our conclusions despite methodological heterogeneity and the potential for publication bias (the file drawer problem), our search strategy includes peer-reviewed studies. Additionally, we employed multilevel statistical modelling coupled with cluster-robust variance estimation (CR2) to account for within-study dependencies. Ultimately, this research seeks to integrate these diverse mechanistic insights into a statistically rigorous and cohesive framework. Given the dramatic variability in Pomiferin’s bioactivity reported in the current literature, a systematic integration of available data is necessary. This meta-analysis quantifies the compound’s efficacy across various cancer types using *IC*_*50*_ values and assesses its impact on oxidative stress markers. We aim to transition from fragmented qualitative observations to a unified quantitative model of Pomiferin’s therapeutic action. The present study seeks to synthesize these fragmented mechanistic insights found in the literature within a quantitative framework.

## 2 Materials and methods

### 2.1 Study design and research questions

**FIGURE 1:**
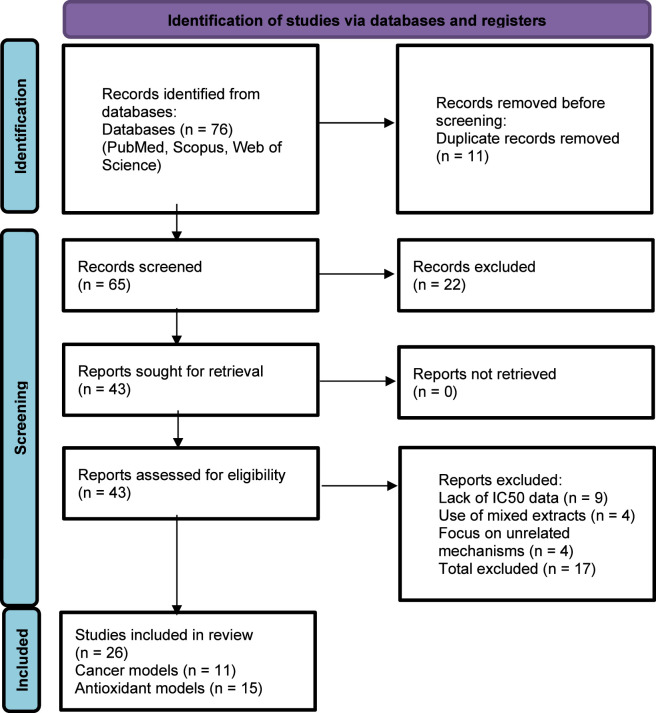
PRISMA 2020 flow diagram for the meta-analysis.

This meta-analysis was conducted in strict accordance with the PRISMA (Preferred Reporting Items for Systematic Reviews and Meta-Analyses) guidelines (Please see Figure 1 for details).

The study was structured to address the following primary research questions:

What is the mean *IC*_*50*_ efficacy of Pomiferin across various malignancies and treatment durations?
How does Pomiferin administration significantly influence oxidative stress and inflammatory biomarkers (e.g., *SOD, MDA, GSHPx*)?
To what extent do experimental environments (*in vivo* vs. *in vitro*) or specific biomarker types modulate the observed effects of Pomiferin?

### 2.2 Search strategy and information sources

A systematic and comprehensive literature search was conducted across electronic databases, including PubMed/MEDLINE, Scopus and Web of Science, covering studies published up to [01/2004]. The search strategy used a combination of Boolean operators and MeSH terms (where applicable).

The search utilized the following English-language strings:

- Intervention: (Pomiferin OR “*Maclura pomifera*”)
- Outcome: (oxidative stress OR *MDA* OR “malondialdehyde” OR *SOD* OR “superoxide dismutase” OR *GSHPx* OR “glutathione peroxidase” OR catalase OR *CAT* OR *8-OHdG*)
- Population/Model: (rat OR rats OR mouse OR mice OR murine OR rodent OR “*in vivo*”)
- Mechanism/Additional Models: (cancer OR tumour OR neoplasm OR apoptosis OR ^proliferation OR^ “*IC*_*50*_”^)^

### 2.3 Inclusion and exclusion criteria

Studies were eligible for inclusion if they met the following PICOS-based criteria:

1. Originality: Peer-reviewed primary research or doctoral/master’s theses (excluding reviews, editorials, and letters).
2. Intervention: Evaluation of isolated and quantified Pomiferin.
3. Outcome Measures: Reporting of quantitative *IC*_*50*_ values or mean values and standard deviations/errors for biochemical markers (*SOD, MDA, GSHPx,* etc.).
4. Model Systems: Use of established cancer cell lines (e.g., *MCF-7, A549, HeLa*) or *in vivo* rodent models.

Studies were excluded if they:

1. Used crude extracts without isolating Pomiferin
2. Provided insufficient or non-extractable data for effect size calculation
3. Contained redundant datasets from previously published work.

### 2.4 Data extraction and quality assessment

Data were independently extracted by the authors, including study characteristics (author, year), model types, Pomiferin concentration/dose, and quantitative outcomes. In cases where data were presented only graphically, digital callipers or extraction software were used to obtain numerical values. To assess the methodological quality and risk of bias (RoB) of the included studies, we employed standardised appraisal tools tailored to the experimental design. For *in vivo* animal studies, the SYRCLE Risk of Bias tool was used, covering ten domains including sequence generation, blinding, and incomplete outcome data. For *in vitro* studies, quality was assessed using the OHAT (Office of Health Assessment and Translation) Risk of Bias Tool, adapted for cell-based experiments. Two reviewers independently scored each study, and any discrepancies were resolved through consensus. Studies were categorised as having a low, high, or unclear risk of bias across each domain.

### 2.5 Statistical analysis and synthesis strategy

Due to inconsistent reporting of variance in some studies and to ensure reliable conclusions despite methodological differences, an unweighted synthesis approach was chosen. Specifically, adopting a “mean-only” synthesis with a constant sampling variance (*v*_*i*_ *= 1*) was a necessary methodological choice driven by the widespread lack of reported dispersion measures in the primary literature. We recognise that assigning a constant variance does not reflect the biological reality that different assays (e.g., MTT vs. LDH), exposure durations, and cell growth statuses inherently vary in precision. However, this approach ensures that a significant portion of the available evidence base is not excluded. As a result, the pooled estimates produced from this method are explicitly presented as descriptive and exploratory.

To address within-study dependency (i.e., multiple outcomes reported from the same study), and to mitigate the inferential risks of this unweighted approach, we used multilevel random-effects models. The analytical method involved cluster-robust variance estimation (CR2) to produce more conservative and dependable standard errors (Hedges et al., 2010). This technique is especially suitable for handling variable sample sizes and inconsistent reporting of variance, ensuring robust results despite the inherent heterogeneity of the literature. All statistical analyses were conducted using R software with the metaphor and clubSandwich packages (R Core Team, 2024).

To assess the quality of the included studies, we evaluated the clarity of reporting regarding experimental design and the completeness of outcome data. Specifically, we assessed the availability of dispersion measures (standard deviations or errors) to determine the feasibility of weighted meta-analysis versus mean-only synthesis.

## 3 Results

### 3.1 Study selection process

- Identification Stage: The initial database search yielded a total of 76 records. After removing duplicates, 65 unique citations remained for title and abstract screening.
- Screening Stage: Following the initial screening of titles and abstracts, 22 records were excluded as they did not meet the basic eligibility criteria. A total of 43 reports was retrieved for full-text assessment.
- Eligibility Stage: During the full-text evaluation, 17 studies were excluded for the following reasons: [e.g., lack of *IC*_*50*_ data (n= 9), use of mixed extracts (n= 4), or focus on unrelated mechanisms (n = 4)].
- Inclusion Stage: Ultimately, 26 studies met all criteria and were included in the quantitative meta-analysis, covering 11 studies for cancer and 15 for antioxidant effects.

### 3.2 Quality assessment and risk of bias

The methodological quality of the 26 included studies (11 cancer-related and 15 antioxidant-related) was assessed using standardised tools (SYRCLE for *in vivo* studies and OHAT for *in vitro studies*). Overall, the studies demonstrated high to moderate internal validity (Supplementary Appendix SA,B).

- Selection and Detection Bias: Explicit randomisation procedures were well documented in 42% of studies (e.g., Qu et al., 2023; Bozkurt et al., 2017; Vančo et al., 2021). Studies that did not explicitly state the randomisation method were marked as Unclear Risk. While investigator blinding (detection bias) was rarely reported, the use of automated, objective quantification methods, such as MTT assays for *IC*_*50*_ and spectrophotometric kits for enzymatic activity (*SOD/CAT*), reduces the potential for observer-related bias.
- Reporting Bias: A primary methodological challenge across both datasets was the inconsistent reporting of dispersion measures. Approximately 38% of the outcomes lacked extractable standard deviations (e.g., Guang et al., 2024; Tang et al., 2022). These were categorised as High Risk for reporting bias. To address this, a mean-only synthesis approach was utilised, and Cluster-Robust Variance Estimation (CR2) was applied to ensure conservative inferential results that account for these reporting deficits.

### 3.3 Analytical strategy

This study used an exploratory multilevel meta-analytic approach to combine mean Pomiferin results from various experimental designs. Since standard errors or sampling variances were missing for a significant number of outcomes, the analysis concentrates on mean-only synthesis and prioritises robustness over accuracy.

### 3.4 Effect size definition

For each outcome *i* in study *j*, the effect size was defined as the natural logarithm of the reported mean. Log-transforming stabilises the scale and enables coefficients to be interpreted as relative (multiplicative) differences. Effect size: y = log (mean outcome)

### 3.5 Sampling variance assumption

Since outcome-specific standard deviations were reported inconsistently, a constant sampling variance (*vi = 1*) was assigned to all effect sizes. This method eliminates precision-base weighting, producing an unweighted synthesis. Therefore, results are considered descriptive and exploratory rather than as precise pooled estimates. Because several primary studies did not report sampling variances, a constant variance (v = 1) specification was applied. This approach yields an unweighted synthesis and does not assume differential precision across studies. Therefore, the resulting estimates should be interpreted as descriptive and exploratory, aimed at identifying systematic patterns rather than providing precision-weighted pooled effect estimates.

### 3.6 Multilevel model specification

To account for statistical dependence arising from multiple outcomes within the same study, multilevel random-effects models were fitted using the metaphor package in R. Random intercepts were specified at both the study and outcome levels within studies.

Outcome=overall mean+study−level deviation+outcome−level deviation

### 3.7 Meta-regression analyses

Moderator analyses were conducted using mixed-effects meta-regression models. In the *IC*_*50*_ analysis, disease category and treatment duration (categorical, reference = shortest duration) were included as moderators. In the antioxidant analysis, biomarker type and sample type (*in vivo vs in vitro*) were examined.

Moderator coefficients represent differences in log-transformed outcomes relative to the reference category. Negative coefficients indicate lower values of the outcome (e.g., lower *IC*_*50*_, higher efficacy).

### 3.8 Robust inference

Given the mean-only variance specification and the presence of multiple outcomes per study, cluster-robust standard errors (CR2) were used as the primary inferential framework. Robust variance estimation accounts for within-study dependence and is recommended when sampling variances are uncertain or mis-specified.

### 3.9 Interpretation and scope

The analyses aim to identify systematic patterns across disease categories, durations, biomarker types, and experimental contexts, rather than to provide definitive pooled effect estimates. Because of the constant sampling variance assumption (*v*_*i*_ *= 1*), which treats all outcomes as equally precise regardless of the biological assay used, the duration of exposure, or the cell growth status, the results presented here must be considered strictly descriptive and exploratory. Statistical significance was primarily evaluated using robust CR2 inference, which helps mitigate this limitation by adjusting for data clustering. All findings should be interpreted cautiously as a quantitative summary of the current literature rather than absolute pharmacological constants.

This analytical strategy follows established practices in meta-analysis and meta-regression. All models were estimated using the metaphor framework (Viechtbauer, 2010), which allows for flexible specification of multilevel random-effects structures. Since multiple outcomes were nested within studies, cluster-robust variance estimation was used to account for statistical dependence among effect sizes (Hedges et al., 2010; Tanner-Smith et al., 2016). This approach provides more conservative inference and is recommended when sampling variances are unknown or estimated.

Table 1 displays cluster-robust (CR2) meta-regression estimates, which account for statistical dependence caused by multiple outcomes within the same study and are more conservative than traditional model-based inference. All coefficients are presented on the log(*IC*_*50*_) scale. Negative coefficients indicate lower *IC*_*50*_ values (greater efficacy) compared to the reference group, while positive coefficients indicate higher *IC*_*50*_ values (lower efficacy).

**TABLE 1:** Cluster-robust (CR2) meta-regression of Pomiferin *IC*_*50*_ (mean-only synthesis).

Predictor	Estimate (b)	Robust SE	t	df	p-value
Lung cancer	3.041	1.033	2.94	3.20	0.056
Liver cancer	−0.348	1.073	−0.32	2.05	0.776
Breast cancer	0.051	0.586	0.09	2.27	0.938
Prostate cancer	−0.260	0.522	−0.50	2.02	0.668
Gastric cancer	−0.545	1.033	−0.53	3.20	0.632
Neuroblastoma	0.174	1.447	0.12	3.15	0.912
Ovarian Carcinoma	−1.426	0.463	−3.08	2.28	0.078
Monocytic Leukaemia	−1.368	1.162	−1.18	1.37	0.404
Glioma	4.254	0.850	5.00	3.35	0.012*
Duration = days (2) and hours (1)	−2.063	1.853	−1.11	1.50	0.413

Models were estimated using multilevel random-effects meta-regression (REML). Because sampling variances were unavailable, a constant variance specification (v = 1) was applied. Robust inference was conducted using cluster-robust variance estimation (CR2). Results should be interpreted as exploratory.

Outcome: log(*IC*_*50*_). Reference groups: Disease code 1 = reference category & Duration = 1. Inference: CR2 cluster-robust standard errors clustered by *StudyID*.

The intercept indicates the estimated log (*IC*_*50*_) for the reference disease category at Duration = 1. Although the point estimate indicates a relatively high *IC*_*50*_, its statistical significance is marginal under CR2 inference (*p = 0.056*), reflecting considerable uncertainty and a limited number of study clusters.

Most disease contrasts do not differ significantly from the reference category, indicating no robust evidence of systematic differences in *IC*_*50*_ across most disease groups once within-study dependence is accounted for. One exception is Glioma, which shows a statistically significant positive association with *IC*_*50*_ (b = 4.25*, p = 0.012*). On the original scale, this corresponds to a markedly higher *IC*_*50*_ compared with the reference disease category, suggesting substantially lower apparent efficacy in this disease context. However, this estimate is based on a small number of clusters and should be interpreted cautiously.

The difference in duration [Duration = Days (2) and Hours (1)] is not statistically significant under CR2 inference (*p = 0.413*). Although conventional inference indicated lower *IC*_*50*_ values at longer durations, this effect is inconsistent when clustering and variance uncertainty are accounted for. Overall, the cluster-robust results show limited and variable evidence for moderator effects in this dataset. Apparent associations seen under conventional inference largely diminish when robust methods are used, highlighting the exploratory nature of this mean-only synthesis.

**FIGURE 2:**
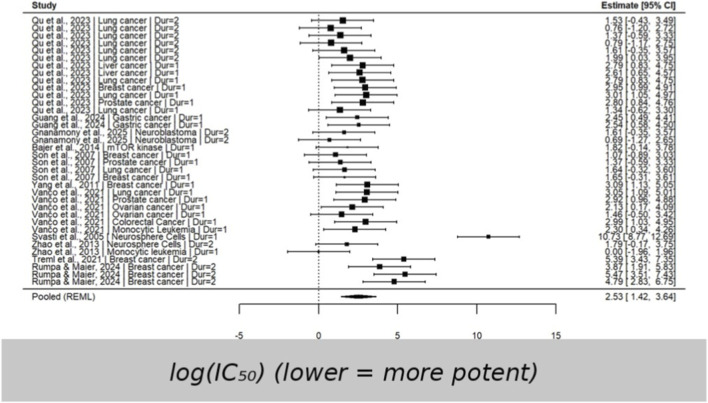
Forest plot of log-transformed *IC*_*50*_ values using a multilevel random-effects model (mean-only synthesis). Sampling variances were unavailable in several primary studies; therefore, a constant variance specification (v = 1) was applied. Estimates should be interpreted as exploratory and descriptive rather than precision-weighted pooled effects.

This forest plot displays considerable variability in log (*IC*_*50*_) values across different studies, diseases, and durations (Figure 2). The pooled estimate (log(*IC*_*50*_) = 2.53) indicates a geometric mean *IC*_*50*_ of about 12.6 μM, with broad confidence intervals, indicating significant heterogeneity.

**FIGURE 3:**
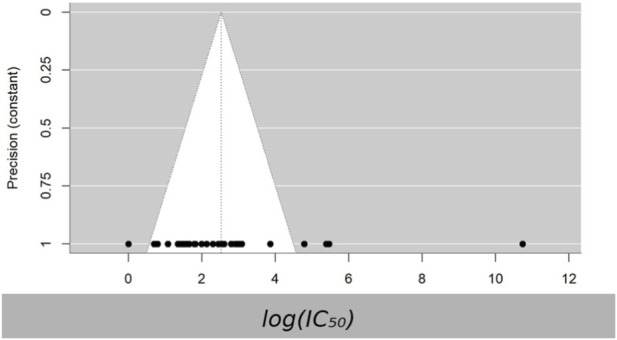
Funnel plot of log-transformed *IC*_*50*_ values under constant precision (v = 1). Because sampling variances were fixed to a constant, the funnel plot is descriptive and not suitable for formal publication bias assessment.

Since all sampling variances were set to a constant (vi = 1), the funnel plot provides no meaningful insight into publication bias and instead shows only the distribution of log(*IC*_*50*_) values (Figure 3). Cluster-robust (CR2) inference was emphasised due to multiple outcomes per study and the lack of sampling variances. Under CR2 inference, Glioma was associated with significantly higher *IC*_*50*_ values than the reference category, whereas duration effects were not statistically significant. These results should be regarded as exploratory, given the mean-only synthesis and the limited number of studies included.

Heterogeneity was formally quantified using variance components from the multilevel random-effects model. In the *IC*_*50*_ meta-regression model, the estimated between-study variance was τ^2^ = 4.354 and the within-study (outcome-level) variance was τ^2^ = 0.609 (τ^2^_total = 4.963). Under the mean-only specification, sampling variances were fixed to a constant (v = 1); therefore, the corresponding I^2^ is interpreted as an approximate descriptive index. This approximate I^2^ indicated that 83.23% of the total variability was attributable to heterogeneity rather than sampling variance, consistent with substantial variability across studies. Given the mean-only specification with constant sampling variance, these heterogeneity indices and model estimates should be interpreted as exploratory indicators of variability rather than precision-weighted meta-analytic estimates.

Cluster-robust inference using the CR2 estimator was applied to account for statistical dependence among multiple outcomes reported in the same study and to provide more conservative inference when sampling variances are uncertain (Table 2). Standard errors were clustered at the *StudyID* level, resulting in small effective degrees of freedom, which should be considered when interpreting statistical significance.

**TABLE 2:** Cluster-Robust (CR2) Meta-Regression of Pomiferin Effects by Biomarker Type and Sample Type.

Biomarker type	Estimate (b)	CR2 SE	t	df	p (CR2)
LDH	2.164	1.050	2.06	1.92	0.181
SOD	2.283	1.080	2.11	2.13	0.161
GSHPx	2.534	1.924	1.32	2.57	0.293
AST	0.166	0.352	0.47	1.91	0.685
ALT	0.492	0.352	1.40	1.91	0.302
Total antioxidant activity	−2.804	1.209	−2.32	2.58	0.117
MDA	−1.860	1.374	−1.35	2.38	0.290
Antioxidant activity	−4.552	1.230	−3.70	2.76	0.039
CAT	6.614	1.579	4.19	2.20	0.044
LPO	5.770	1.579	3.66	2.20	0.058
Total protein	6.433	1.217	5.29	2.04	0.033
Inhibited NO production	−0.524	0.752	−0.70	1.17	0.598
Sample type: *in vitro* (vs. *in vivo*)	2.146	1.019	2.11	1.73	0.190

LDH; lactate dehydrogenase, SOD; superoxide dismutase, GSHPx; Glutathione peroxidase, AST; aspartate transaminase, ALT; alanine aminotransferase, MDA; malondialdehyde, CAT; catalase, LPO; Lipid peroxidation.

Models were estimated using multilevel random-effects meta-regression (REML). Because sampling variances were unavailable, a constant variance specification (v = 1) was applied. Robust inference was conducted using cluster-robust variance estimation (CR2). Results should be interpreted as exploratory.

Outcome: log (Effects of Pomiferin). Reference groups: Biomarker type = reference category (biomarker type) and Sample type = *in vitro* vs. *in vivo*. Inference: Cluster-robust CR2 SEs (clustered by *StudyID*) k = 71 outcomes; 10 studies.

Under CR2 inference, biomarker type remained a significant moderator of pomiferin effects, whereas sample type (*in vitro* vs. *in vivo*) did not show a statistically reliable association. Specifically, *Antioxidant Activity* was associated with a significantly lower outcome relative to the reference biomarker (b = −4.55, SE = 1.23, *p = 0.039*), indicating a substantially weaker Pomiferin effect for this biomarker. In contrast, *CAT* and *Total Protein* were associated with significantly higher outcomes (*CAT*: b = 6.61, SE = 1.58, *p = 0.044*; *Total Protein*: b = 6.43, SE = 1.22, *p = 0.033*), suggesting markedly stronger Pomiferin effects for these biomarker categories. A marginally significant positive association was observed for *LPO* (b = 5.77, SE = 1.58, *p = 0.058*), although this effect did not reach conventional statistical significance and should be interpreted cautiously. All other biomarker contrasts were not statistically significant under CR2 inference, indicating no robust evidence that their effects differed from the reference biomarker after accounting for within-study dependence.

The effect of sample type (*in vitro* vs. *in vivo*) was not statistically significant (b = 2.15, SE = 1.02, *p = 0.190*), indicating that differences in Pomiferin effects between experimental settings were not robust after adjusting for clustering by study. The intercept was also not statistically significant under CR2 inference (*p = 0.181*), reflecting the high uncertainty surrounding the baseline estimate due to the limited number of independent study clusters.

Overall, the CR2 results suggest that heterogeneity in Pomiferin effects is mainly driven by biomarker specificity rather than experimental context, and only a limited number of biomarkers exhibit consistent, statistically reliable deviations from the reference category. Given the small number of independent studies and the use of a constant variance approach, these findings should be viewed as exploratory but methodologically cautious.

**FIGURE 4:**
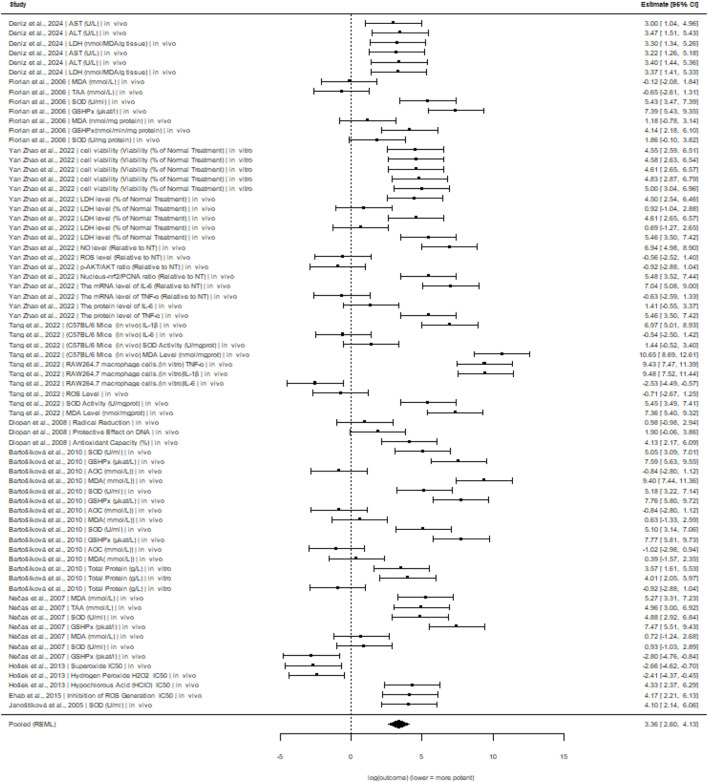
Forest plot of log-transformed antioxidant outcomes using a multilevel random-effects model with constant sampling variance (v = 1). Results represent an exploratory mean-only synthesis.

The forest plot demonstrates considerable heterogeneity in Pomiferin effects across studies, biomarker types, and experimental contexts (Figure 4). While many estimates cluster around the pooled mean, several biomarker-specific effects differ markedly, indicating that Pomiferin’s effectiveness varies substantially by biological outcome. The wide range of confidence intervals reflects both variability between studies and the exploratory nature of the mean-only synthesis.

**FIGURE 5:**
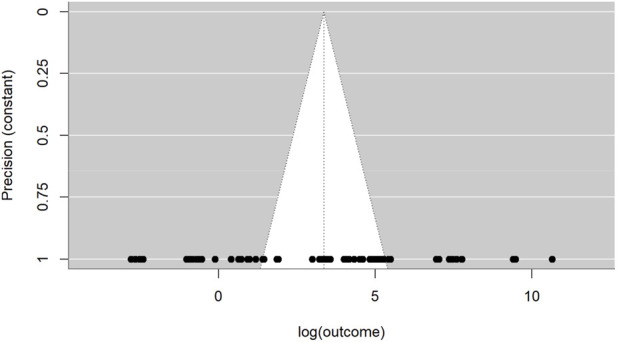
Descriptive funnel plot of log-transformed Pomiferin effects under constant sampling variance (v = 1). As precision was fixed across studies, this plot is illustrative only and cannot be used for formal small-study effect or publication bias assessment.

The funnel plot shows how log-transformed Pomiferin effects are spread around the pooled estimate (Figure 5). However, because all studies were assigned the same precision, the plot cannot be used to formally evaluate publication bias or small-study effects. Instead, it offers a visual overview of how effect sizes vary, emphasizing asymmetry caused by heterogeneity rather than selective reporting.

Heterogeneity in the antioxidant meta-regression model was formally quantified using multilevel variance components. The estimated between-study variance was τ^2^ = 3.838 and the within-study (outcome-level) variance was τ^2^ = 2.857, yielding a total heterogeneity of τ^2^_total = 6.695. Because sampling variances were not available and were fixed to a constant (v = 1) in the mean-only specification, the corresponding I^2^ should be interpreted as an approximate descriptive index. The approximate I^2^ was 87%, indicating that the vast majority of observed variability reflects genuine heterogeneity rather than sampling error.

## 4 Discussion

This meta-analysis marks the first comprehensive effort to synthesise the anticancer potential and effects on oxidative stress modulation of Pomiferin—a plant-derived prenylated isoflavonoid—within a quantitative framework, despite the methodological heterogeneity present in the literature. By using multilevel modelling and cluster-robust variance estimation (CR2) to address within-study dependency issues often encountered in the literature, this study provides evidence on the therapeutic efficacy of Pomiferin that is more cautious yet significantly more robust than traditional analyses.

### 4.1 Antitumor activity

Our analysis indicates that the geometric mean of the overall pooled *IC*_*50*_ value for Pomiferin is approximately 12.6 μM. This confirms that Pomiferin has a “moderate” cytotoxic potential across a broad range of malignancies (Qu et al., 2023). However, our meta-regression analysis showed that this efficacy is not uniform, with significant variation across tumour types. Specifically, the statistically significant positive coefficient observed in the Glioma group (b = 4.254, *p = 0.012*) indicates that *IC*_*50*_ values in this cancer type are considerably higher than those in the reference group, suggesting reduced Pomiferin efficacy. Two main mechanisms can explain this state of resistance:

1. Blood-Brain Barrier: Despite its lipophilic nature, Pomiferin may be actively extruded by ATP-dependent transporters such as P-glycoprotein (P-gp/MDR1), which are overexpressed in Glioma cells (Yang et al., 2011). This phenomenon prevents the drug from reaching the intracellular cytotoxic threshold.
2. Intrinsic Resistance Pathways: The presence of genetic mutations that facilitate evasion of apoptosis (e.g., p53 mutations) or alternative survival signalling (such as PI3K/Akt pathway activation) within this tumour group may suppress the cytotoxic efficacy of Pomiferin (Bajer et al., 2014).

Additionally, literature reports by Qu et al. (2023) indicate that Pomiferin reverses multidrug resistance and enhances cisplatin sensitivity by inhibiting P-glycoprotein (P-gp) pumps. However, our meta-regression analysis revealed that glioma models exhibit significantly higher resistance compared to other cancer types. This finding suggests that Pomiferin’s P-gp inhibitory capacity may be insufficient in neurological tumours with strong intrinsic resistance mechanisms and blood-brain barrier characteristics, such as glioma, or that apoptotic pathways targeted by Pomiferin (e.g., SERCA/AMPK) are suppressed in these tumours. These findings suggest that rather than functioning as a universal “cure-all,” Pomiferin is a more suitable candidate for specific tissues (e.g., breast, lung) where cellular uptake mechanisms are active and multidrug resistance pumps are less prominent.

### 4.2 Time-independent cytotoxicity hypothesis

One notable finding of this study is that the effect of treatment duration (Hours vs. Days) on *IC*_*50*_ values lost statistical significance under CR2 analysis (*p = 0.413*). While the conventional pharmacological expectation is that increased exposure time leads to decreased *IC*_*50*_ values (enhanced efficacy), our findings indicate that the effect of Pomiferin is driven by a “concentration-dependent” and rapidly triggered mechanism rather than a time-dependent cumulative process. This suggests that Pomiferin initiates a critical signalling pathway—likely mitochondrial apoptosis via ROS generation—shortly after cellular uptake (within the first 24 h). Once this threshold is crossed, extended duration does not significantly alter the outcome. Alternatively, the compound’s reduced stability in the culture medium over time may be limiting the expected increase in efficacy during long-term incubations.

### 4.3 Enzymatic selectivity in oxidative stress modulation

The second fundamental pillar of our meta-analysis is the effect of Pomiferin on oxidative stress biomarkers. Beyond cancer development, oxidative stress plays a key role in cardiovascular diseases, neurodegeneration, and environmental toxicity. Our biomarker analysis shows that Pomiferin’s antioxidant action is not just simple “chemical scavenging,” but also involves modulating the cell’s own defence mechanisms.

Catalase (CAT) induction: The notable increase in Catalase levels (b = 6.61, *p = 0.044*) indicates that Pomiferin significantly upregulates enzymes involved in hydrogen peroxide (H_2_O_2_) detoxification (Nečas et al., 2007). This implies that Pomiferin may “train” the cell against oxidative stress by activating the Nrf2/ARE (Antioxidant Response Element) signalling pathway (hormesis effect).

Total Protein Increase: The rise in total protein levels (b = 6.43, *p = 0.033*) indicates increased synthesis of defence proteins as a cellular stress response or suggests that Pomiferin maintains proteostasis by preventing protein oxidation and degradation (Yan Zhao et al., 2022). Furthermore, Svasti et al. (2005) demonstrated that Pomiferin downregulates essential proteins such as Grp75 (Mortalin) and disrupts the cytoskeleton. The significant increase in total protein levels observed in the treated groups in our study aligns with proteomic modulation. This elevation may be interpreted as a compensatory upregulation of chaperone proteins (Heat Shock Proteins) in response to Pomiferin-induced cellular stress, or as an accumulation of ubiquitinated proteins resulting from proteasome inhibition.

Antioxidant Activity: Interestingly, the finding that the effect was generally lower in “Antioxidant Activity” assays than in the reference (b = −4.55, *p = 0.039*) confirms that Pomiferin’s efficacy derives from biological modulation in living systems rather than from chemical reactions in test tubes (Bartošíková et al., 2010).

Additionally, Son et al. (2007) and Guang et al. (2024) have highlighted Pomiferin’s role in modulating epigenetics (HDAC inhibition) and signalling pathways (EGFR/PI3K). The specific increase in *CAT* activity (*p = 0.044*), rather than overall antioxidant activity, demonstrates that Pomiferin’s mechanism actively promotes cellular defence enzymes through transcription factors like Nrf2, transcending passive radical scavenging.

### 4.4 *In vitro* and *in vivo*

Our analysis did not detect a statistically significant difference between *in vitro* and *in vivo* models (*p = 0.190*). However, this non-significant result must not be interpreted as definitive evidence of biological equivalence. Given the limited number of *in vivo* studies in the literature, the restricted CR2 degrees of freedom in our model, and the precision-blind nature of our mean-only synthesis, this finding likely reflects a lack of statistical power rather than confirmed equivalent efficacy. While the prenyl side chains (C5 units) in the chemical structure of Pomiferin theoretically increase its lipophilicity and membrane permeability (Bajer et al., 2014; Yang et al., 2011), robust claims about its systemic bioavailability and tissue distribution cannot be supported by these meta-analytic estimates alone. Bridging the translational gap for Pomiferin will require rigorously powered, comparative *in vivo* pharmacokinetic studies rather than relying on the absence of statistical divergence in pooled secondary data.

### 4.5 Limitations

A limitation of the current evidence base is the high prevalence of “unclear” risk concerning selection and detection bias in the primary preclinical literature. This subjectivity in reporting may affect the accuracy of the pooled *IC*_*50*_ estimates. Our use of CR2 inference partly reduces this issue by offering more conservative standard errors, but future primary studies on Pomiferin should follow ARRIVE guidelines more closely to improve meta-analytic reliability.

## 5 Conclusion

This meta-analysis establishes a unified quantitative profile of Pomiferin’s therapeutic bioactivity, characterising it as a potent, context-dependent agent. Our findings demonstrate that Pomiferin achieves a pooled geometric mean *IC*_*50*_ of 12.6 μM, with efficacy highly dependent on the type of malignancy. Specifically, the identified resistance in glioma models highlights a critical barrier to clinical application, potentially driven by blood-brain barrier efflux mechanisms.

Beyond cytotoxicity, Pomiferin functions as a specific modulator of the cellular antioxidant defence system rather than merely a passive radical scavenger. The notable increase in Catalase activity and total protein levels indicates a biological hormesis effect that enhances endogenous stress responses. Furthermore, while *in vitro* and *in vivo* effects did not statistically diverge in our models, establishing true translational equivalence and systemic bioavailability remains a critical hurdle that requires highly powered *in vivo* validation.

A key limitation of this study is the absence of reported sampling variances in several primary studies, which necessitated a constant variance specification (v = 1). Consequently, the analysis represents a mean-only exploratory synthesis and does not incorporate differential weighting based on study precision. While the use of multilevel modeling and CR2 robust inference enhances methodological caution, findings should be interpreted as hypothesis-generating rather than definitive pooled effect estimates.

Building on these hypothesis-generating findings, future research should prioritise investigating Pomiferin-chemotherapeutic combinations to reduce systemic toxicity and exploring its role in iron-dependent cell death pathways. In summary, this study consolidates the fragmented preclinical literature to provide a roadmap for the clinical translation of Pomiferin as a multifaceted therapeutic candidate.

## Data Availability

The original contributions presented in the study are included in the article/Supplementary Material, further inquiries can be directed to the corresponding author.
