# Supplementary material for: Unveiling the therapeutic profile of pomiferin: a meta-analysis of cytotoxicity and oxidative stress modulation in preclinical models
Source: Front Pharmacol. 2026 Mar 23;17:1789772. doi: 10.3389/fphar.2026.1789772 (PMC13050899; doi:10.3389/fphar.2026.1789772)
Supplement: Supplementary file 1 [file Supplementaryfile1.docx]

Appendix A

| **Study (ID)** | **Model** | **Selection Bias** | **Detection Bias** | **Attrition Bias** | **Reporting Bias** | **Quality** |
| --- | --- | --- | --- | --- | --- | --- |
| Qu et al. | Mixed | Low Risk | Unclear | Low Risk | Low Risk | High |
| Guang et al. | Mixed | Unclear | Unclear | Low Risk | High Risk | Moderate |
| Gnanamony | In vitro | Unclear | Unclear | Low Risk | Low Risk | High |
| Bajer et al. | In vitro | Unclear | Unclear | Low Risk | High Risk | Moderate |
| Son et al. | In vitro | Low Risk | Unclear | Low Risk | Low Risk | High |
| Yang et al. | In vitro | Unclear | Unclear | Low Risk | Low Risk | High |
| Vančo et al. | In vitro | Low Risk | Unclear | Low Risk | Low Risk | High |
| Svasti et al. | In vitro | Unclear | Unclear | Low Risk | High Risk | Moderate |
| Zhao et al. | In vitro | Unclear | Unclear | Low Risk | Low Risk | High |
| Treml et al. | In vitro | Unclear | Unclear | Low Risk | High Risk | Moderate |
| Rumpa et al. | In vitro | Low Risk | Unclear | Low Risk | Low Risk | High |

Appendix B

| **Study** | **Model** | **Selection Bias (Randomization)** | **Detection Bias (Blinding)** | **Attrition Bias (Missing Data)** | **Reporting Bias (Missing SD/Var)** | **Overall Quality** |
| --- | --- | --- | --- | --- | --- | --- |
| Deniz et al. (2024) | In vivo | Unclear | Unclear | Low Risk | **High Risk** (LDH missing SD) | Moderate |
| Florian et al. (2006) | In vivo | Low Risk* | Unclear | Low Risk | Low Risk | High |
| Yan Zhao et al. (2022) | In vitro | Unclear | Unclear | Low Risk | Low Risk | High |
| Tang et al. (2022) | Mixed | Unclear | Unclear | Low Risk | **High Risk** (Multiple missing SD) | Moderate |
| Diopan et al. (2008) | In vitro | Unclear | Unclear | Low Risk | **High Risk** (Missing SD) | Moderate |
| Bartošíková et al. (2010) | In vivo | Low Risk* | Unclear | Low Risk | Low Risk | High |
| Nečas et al. (2007) | In vivo | Low Risk* | Unclear | Low Risk | Low Risk | High |
| Hošek et al. (2013) | In vitro | Unclear | Unclear | Low Risk | Low Risk | High |
| Ehab et al. (2015) | In vitro | Unclear | Unclear | Low Risk | Low Risk | High |
| Janoštíková et al. (2005) | In vivo | Low Risk* | Unclear | Low Risk | Low Risk | High |
| Nešuta et al. (2011) | In vitro | Unclear | Unclear | Low Risk | Low Risk | High |
| Vesela et al. (2003) | In vitro | Unclear | Unclear | Low Risk | Low Risk | High |
| Bozkurt et al. (2017) | In vivo | Low Risk* | Unclear | Low Risk | Low Risk | High |
| Tsao et al. (2003) | In vitro | Unclear | Unclear | Low Risk | Low Risk | High |

**Studies rated "Low Risk" for Selection Bias typically specify standardized animal groupings or explicit randomization in their methodology.*
